# Health and social services integration in the Veneto Region

**Published:** 2012-09-04

**Authors:** C Saccavini, S Mancin, A Favaro

**Affiliations:** Consorzio Arsenàl, Italy; Consorzio Arsenàl, Italy; Consorzio Arsenàl, Italy

**Keywords:** telemonitoring, chronic diseases, frail people, social and health services integration

## Abstract

**Purpose:**

Veneto Region is currently achieving the integration of health and social services by integrating the tele-surveillance (detection of emergencies) already provided to ‘socially frail’ people with tele-monitoring, addressed to patients affected by chronic diseases.

**Theory:**

This strategy is guided by the idea that a better integration between the different levels of assistance is capable of improving the quality and continuity of care and generating synergies that benefit the healthcare system both by enhancing coordination of efforts and by reducing costs.

**Methods:**

The strategy employs a unique call center at Regional level, that currently performs tele-surveillance services (a social service) originally targeted to ‘socially frail’ people and a more complete tele-monitoring service for chronic patients, thus integrating health and social services. Tele-monitoring consists in the remote measurement of vital parameters (controlled by physicians), and the management of emergencies.

**Results and conclusions:**

This approach to the integration of different assistance levels is being put to the test in the context of the RENEWING HEALTH European Project. The project will assess the services using a multidisciplinary HTA methodology thus providing reliable evidence on the effectiveness and cost-effectiveness on the large-scale implementation of this kind of service.

Powerpoint presentation available at http://www.integratedcare.org at congresses – San Marino – programme.

